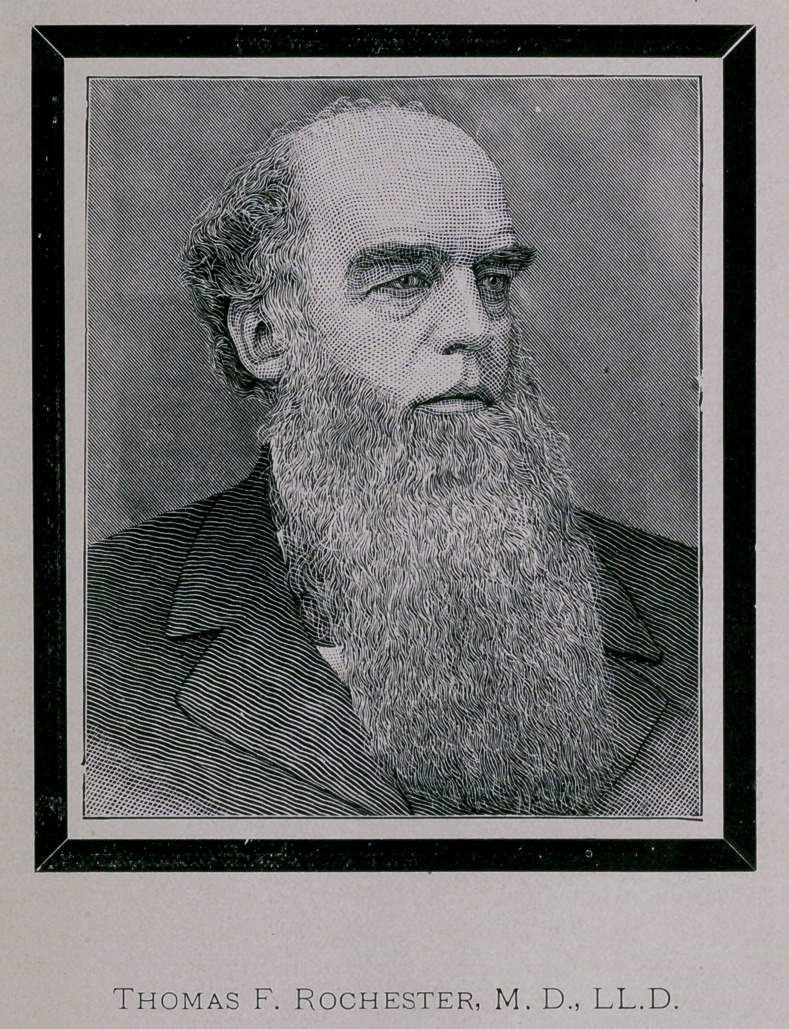# Thomas F. Rochester, M. D., LL.D.

**Published:** 1887-06

**Authors:** 


THOMAS F. ROCHESTER, M. D., LL.D.

### Obituary.

*THOMAS F. ROCHESTER, M. D., LL.D.*

One year ago, after a partial recovery of Dr. Rochester from a long and painful illness, the JOURNAL paid a tribute to his worth and expressed the hope that the noble man and great physician would be spared to this community. But such was not to be, and great is the loss to the profession, to the public, and greater still to the college in which he was the main support. By his death the profession loses a man whose place can scarcely be filled. The public sustains a great loss, the college a loss which years alone and patient labor on the part of the whole faculty can repair. Dr. Rochester maintained his exalted position at the head of the profession, first, because his education was broad and far-reaching. His heart was in his work He continued to be a thorough student and kept abreast with the best scientific work of his time. Thus he feared no rivals. The public estimation, as given in the following extracts from the press and the resolutions of various organizations, show an exalted opinion of the man, which is not greater than ours:

"After a protracted fight against all-conquering Death, he passed away May 24, 1887. Hundreds of friends will feel a sense of personal loss in the demise of this excellent man, who had won for himself a foremost place in the ranks of his profession, who had carved for himself a niche in the Temple of Fame. Not only in his profession was he known and honored, for his name had long been identified with the city's best interests. He was not unknown among the poor and deserving, for his acts of unostentatious charity were many and far-reaching. None knew him but to esteem him; his friendship was valued by all classes.

"The fatal illness may be traced back to an accident which occurred to Dr. Rochester on February 17, 1885, when he was thrown from his cutter while out making professional visits, and seriously injured. The shock to his system caus d him to be confined to his house several weeks. He was subsequently able to be about, but after a few weeks began to be troubled with fierce attacks of asthma, frequently repeated. In April, 1885, he went to Washington, Richmond, and Houghton Row, and came back somewhat relieved. But he was troubled with asthma more or less during the summer and fall, at the same time continuing his professional duties and taking his own remedies. He went to New York to attend a medical meeting on November 20, 1885, and while there suffered a severe attack of asthma, and came back home without completing the business for which he had gone. For four months he was confined to his home. He experienced attacks of asthma almost every day in spite of all efforts, and in December it was also found that he had water in his chest. For this he operated on December 16th, and was relieved of asthma, which continued to trouble him only when water accumulated, especially during the next three months. His health was improved in March, 1886, to such an extent that it was thought a Southern trip would benefit him. While away he submitted to an operation once to relieve him from water. He returned the latter part of May. He had slight accumulation two or three times shortly after this, but eventually was entirely relieved for about two months, and was considered well enough to resume his professional visits to a moderate extent, and commenced his lectures before the Buffalo Medical College. The summer and fall passed away nicely with him. As spring approached he began to feel the effects of his work and was tapped two or three times on account of accumulation of water. Finally in March last the accumulations and consequent tappings had grown much more frequent, and there was a noticeable decline in the patient's constitution, with growing weakness and loss of appetite. The last period of his confinement at home dates from April 12th. For two weeks he did not leave the house. Then he recovered a little and occasionally took a ride out. On the night of May 5th he went to the Buffalo Club. Returning home he fatigued himself greatly in a rapid walk to avoid a storm which was coming on. The next Sunday morning, May 8th, he took a ride, at the close of which he was taken violently ill, wtth an attack of vomiting and symptoms of great prostration. There was no fever, but heavy perspiration, and that irregularity and rapidity of pulse that indicated a failing system. He rested badly that night, but was easier on Monday, and since then had improved only in the way of physical comfort, his pulse varying from 100 to 140.

" His sickness is supposed to have been a chronic inflammation of the kidneys, closely allied to rheumatic or gouty kidney

" Thomas Fortescue Rochester was descended from colonial English settlers of Virginia, eldest son of T. H. and P. E. Rochester, and grandson of Col. Nathaniel Rochester, Deputy Commissary-General in the Continental Army, and for whom the city of Rochester is named.

" In the earlier years of Dr. Rochester's practice in Buffalo he was in partnership with Dr. Austin Flint, Sr. Most of the older practitioners of Buffalo with whom Dr. Rochester was associated are dead. A few remain, an ornament to their profession and an honor to their city. It was a worthy place that Dr. Rochester soon took among such able men as Drs. James P. White, Barnes, Pratt, Charles Winne, Loomis, P. H. Strong. Samo, James and George Hadley, Sanford B Hunt, Charles A. Lee, John C. Dalton, Sanford Eastman, William Ring, and others. Especially in the State Medical Society were Dr. Rochester's professional associations agreeable and eminent. Of Dr. Rochester's professional publications may be mentioned 'The Winter Climate of Malaga,' being observations made during personal residence; 'History of the Medical Societies of Buffalo;' 'The Army Surgeon;' 'The Modern Hygea;' 'Medical Men and Medical Matters of 1776,' and many monographs on various professional subjects.

" Some of the foregoing are of no little local interest. With No. 2 of the first volume of the *Buffalo Medical and Surgical Journal and Reporter*, September, 1861, Dr. Rochester began the publication of a series of articles on the 'History of the

Origin and Transactions of the Medical Societies of Buffalo,' which was continued tnrough three numbers. These papers are probably the best record of the early years of the society ever made. He brought the abstract of proceedings down to April, 1861, beginning with 'the earliest record of medical association for professional improvement and advancement,' which was entitled 'The Constitution and By-Laws of the Medical Society of the Village of Buffalo, adopted July 16, 1831.' The first officers of the association were: President, Dr. Cyrenius Chapin; Vice-President, Dr. Judah Bliss; Recording Secretary, Dr. Bryant Burwell; Corresponding Secretary, Dr. Josiah Trowbridge; Treasurer, Moses Bristol.

"For many years the *Buffalo Medical Journal* contained frequent contributions from Dr. Rochester. Notable among these were papers on cholera, in which are recorded many interesting facts relating to the epidemic of 1854, with observations on cases at Suspension Bridge, Niagara Falls and Buffalo.

"Dr. Rochester was identified with various public institutions. He was President of the Buffalo Fine Arts Academy, and a practical patron and promoter of art. He was also a life member of the Young Men's Association and a member of Trinity Episcopal Church.

"As an instructor he was direct in style, always clear and forcible, aiming to teach, to inculcate a truth, rather than to appear learned in the eyes of his hearers. As a speaker he had often been heard in public. His annual remarks to the graduating classes from the nurses' training school, in connection with the General Hospital, are well remembered as models of kindly, advisory address.

"Beside the widow he leaves six children to grieve at the loss of a kind father and a considerate friend—Mr. Nathaniel Rochester, cashier of the Third National Bank; Dr. Delancey Rochester, and Miss Elizabeth, Miss Margaret, and Miss Emily Rochester, and Mrs. Charles B. Wheeler."

Dr. DeLancey has been with him several years in business, has many of the qualities of his father, and will undoubtedly succeed to his large practice. By natural attainments and by education, as well as by heredity, he is certainly deserving of it.

The Erie County Medical Society met to take action and express their great grief at his loss.

Dr. John D. Hill was chosen chairman and Dr. William H. Thornton secretary. It is to be noted that every member, without exception, who addressed the society upon their common loss, betrayed emotion in voice and manner. Said Dr. Hill:

" Gentlemen of the Erie County Medical Association: You are all aware of the sad event which has called us together. Death has again entered our ranks, and has taken from our galaxy a bright and shining light—may I not say the *savant* of our profession?

" It is fitting on such an occasion that we should meet to pay the tribute and respect due a member who has devoted the energies of a life in advancing the best interests not alone of his chosen avocation, but of the community in which he lived. No member of this society was better known or more highly esteemed than was Dr. Thomas F. Rochester. We do credit to ourselves by honoring his memory."

Dr. Hill then named as a committee to prepare a suitable memorial: Drs. Charles Cary, John Cronyn and J. B. Andrews, who retired for that sad duty.

The committee offered the following memorial:

" After an illness patiently borne for nearly two years, an illness which was heroically but unsuccessfully combatted, Dr. Thomas F. Rochester has been removed from our centre by death. His professional achievements, his absolute integrity, his sound judgment, his profound erudition, his purity of character, and his widespread generosity have endeared him to us all, and raised him to the very summit of distinction in this community; his many virtues have been the means whereby he has attained his brilliant success, and have gained for him the almost unprecedented regard and love of his fellow men, while by the radiance of his character he has invited emulation.

" We recognize that by the death of Dr. Rochester the Erie County Medical Society, the profession, our charities, arts and sciences, have lost a friend and supporter who cannot be replaced; that this society particularly will mourn the loss of one of its oldest, most zealous and able members, whose high attainments have given prominence to this society in the State; that the profession loses an endeared friend from whose source of ever-flowing knowledge we have long been accustomed to draw; our charities have lost a too liberal contributor and a wise adviser, and society an intellectual, courteous and respected member, whose example of cheerfulness and Christianity will outlive his generation.

"*Resolved*, That this memorial be entered upon our minutes, and that a copy be transmitted to his bereaved family, who are not alone in their mourning, but whose terrible grief is shared by this society."

Dr. M. D. Mann, in moving the adoption of the resolution, said that a truly good man had gone; great in soul and mind, with no malice or uncharitableness in his heart; full of sympathy, broad in learning—a Christian in the fullest sense of the word.

Dr. W. W Potter, in seconding the motion, said that not in this century would the society be called upon to mourn the loss of another so great a healer. He knew the deceased for a third of a century as teacher, friend, and neighbor, and in all of these relations he fulfilled the speaker's ideal. The resolution was then unanimously adopted.

Dr. George N. Burwell, who attended Dr. Rochester in his last illness, was unable from emotion to read the tribute he had prepared and the secretary read the following:

"I come to-night, my friends and brethren, to mourn with you the loss of our friend, associate, and exemplar, Dr. Rochester, as well as to add to your tribute of high and most merited praise, the humble assurance of my affection and admiration. During all of his professional life in Buffalo we have worked side by side; always, I am most happy to say, in good understanding, and, therefore, always in peace and the most perfect harmony. He was the younger of the two, and perhaps the more ambitious; certainly the better worker and the more of the man of affairs. So to a certain extent we have worked on different lines; but never, on any occasion, to cross purposes. His push and energy have always claimed, and have had, admiration; and his prosperity and great reputation and renown have been always to me, as a citizen of Buffalo, objects of honest pride. I, therefore, do most sincerely mourn his death as a public loss to the city; and to the gentlemen associated with him in many and various associations it must be almost a calamity.

To myself, let me say, it is an irreparable loss, for it takes from me the one most dear to me, and alas! almost the last one of the associates of my early medical life. His place, his esteem and friendship, which I ever so highly valued, can never be made good to me, and I mourn him as one without hope."

For the beautiful portrait which we are able to present we are under obligations to the *Buffalo Morning Express.*

## Figures and Tables

**Figure f1:**